# Influence of a 10-Day Mimic of Our Ancient Lifestyle on Anthropometrics and Parameters of Metabolism and Inflammation: The “Study of Origin”

**DOI:** 10.1155/2016/6935123

**Published:** 2016-06-06

**Authors:** Leo Pruimboom, Begoña Ruiz-Núñez, Charles L. Raison, Frits A. J. Muskiet

**Affiliations:** ^1^Natura Foundation, 3281 NC Numansdorp, Netherlands; ^2^Laboratory Medicine, University Medical Center Groningen (UMCG) and University of Groningen, 9713 GZ Groningen, Netherlands; ^3^Department of Psychiatry, College of Medicine, John and Doris Norton School of Family and Consumer Sciences, Tucson, AZ 1075, USA

## Abstract

Chronic low-grade inflammation and insulin resistance are intimately related entities that are common to most, if not all, chronic diseases of affluence. We hypothesized that a short-term intervention based on “ancient stress factors” may improve anthropometrics and clinical chemical indices. We executed a pilot study of whether a 10-day mimic of a hunter-gatherer lifestyle favorably affects anthropometrics and clinical chemical indices. Fifty-five apparently healthy subjects, in 5 groups, engaged in a 10-day trip through the Pyrenees. They walked 14 km/day on average, carrying an 8-kilo backpack. Raw food was provided and self-prepared and water was obtained from waterholes. They slept outside in sleeping bags and were exposed to temperatures ranging from 12 to 42°C. Anthropometric data and fasting blood samples were collected at baseline and the study end. We found important significant changes in most outcomes favoring better metabolic functioning and improved anthropometrics. Coping with “ancient mild stress factors,” including physical exercise, thirst, hunger, and climate, may influence immune status and improve anthropometrics and metabolic indices in healthy subjects and possibly patients suffering from metabolic and immunological disorders.

## 1. Introduction

Chronic noncommunicable diseases (CNCD), including diabetes type 2, cardiovascular diseases, autoimmune diseases, chronic fatigue, depression, and neurodegenerative diseases, are the major causes of morbidity, work absence, and invalidity. They may be responsible for 35 million out of 52 million annual deaths worldwide [1]. CNCD have recently become the major topic for the World Health Assembly. In 2013, The Lancet issued a special on CNCD [2, 3]. Treating patients with CNDC is complex and of limited availability in many countries because of costs, while their proximate treatment has many side effects. Compliance is often low (e.g., lipid lowering drugs and antihypertensives) and many interventions have proven unsuccessful [4].

The vast majority of CNCD are caused by unhealthy lifestyle and other anthropogenic factors. It seems that none of us is immune to the damaging effects of the modern lifestyle [5, 6]. Not surprisingly, many of these diseases are preventable by changes in behavior, including nutrition, physical activity, and coping strategies [7–10]. The anthropogenic factors responsible for the CNCD pandemic include physical inactivity, unhealthy diet (e.g., high energy density refined food and too low vegetables, fruits, and fish), chronic psychoemotional stress, insufficient sleep (loss of biorhythm), and environmental toxins, including smoking [5, 11–18]. All of these may be considered “danger signals” that activate central stress axes (sympathetic nervous system (SNS) and hypothalamus-pituitary-adrenal (HPA)) and the immune system [19, 20].

Inflammation is characterized by the five classical symptoms of rubor, dolor, calor, tumor, and torpor. Inflammation requires metabolic adaptations [12]. Overt injury and infection bring about short-term allostatic responses aiming at the removal of the infectious agent, engaging in repair, and, ultimately, recovering homeostasis in a highly coordinated fashion [21]. While ancient infectious challenges induce optimal responses with self-resolving capacity, anthropogenic inflammatory stimuli deriving from modern society provide us with weak and long-lasting immunological responses that take us to a condition of chronic systemic low-grade inflammation with accompanying chronic hypometabolic adaptations [12, 22, 23]. It has become clear that many, if not all, CNCD are characterized by a state of low-grade inflammation (LGI [24, 25]) that comes along with, for example, insulin resistance, hyperleptinemia, cortisol resistance, subclinical hypothyroidism, and nerve-driven immunity. Jointly, they are responsible for the gradual development of multiple comorbidities together referred to as the typically Western diseases of affluence [11, 26–31].

The absence of ancient immune challenges in current Western societies inspired us to hypothesize that acute stress from ancient danger signals causes redistribution of the immune system towards its evolutionary preferred locations and thereby favorably affects the state of chronic systemic low-grade inflammation, normalizes stress axes activities, recovers rhythmic function, and restores insulin-insensitive pathways. Mild stress factors may activate resolution responses based on survival mechanisms that originate from millions of years of evolutionary pressure. In this study we investigated whether such “ancient stressors,” provided by a 10-day trip through the Pyrenees, improved anthropometrics and various clinical chemical parameters of low-grade inflammation, stress, and metabolic control in 55 apparently healthy adults. The objective was to provide proof of principle through the notion that humans can influence their immune and metabolic systems by exposure to ancient mild acute stress factors. The intervention in our pilot study mimicked, to some extent, the “conditions of existence” of ancestral and current hunting/fishing-gathering populations.

## 2. Subjects and Methods

### 2.1. Study Group

The participants were students, scientists, physicians, and other health professionals who were engaged in clinical Psychoneuroimmunology (PNI) courses throughout Europe. They were interested to experience the impact of ancient lifestyle on their own health and well-being and therefore jointly decided to engage in this study. No consent from a medical ethical committee was deemed necessary, for which we refer to the constitutional law of self-determination, in which people have a basic right to decide what they want to do with their health (included in the patients self-determination act, United States 1991 and the Council of Europe 2009). The participants were part of their own team, united in a research consortium (see Acknowledgments). They covered their own expenses and there were no grants. They appointed one of us (LP) as the study coordinator. All volunteers signed an informed consent and all were informed about the trip details. The Catalan Government and the local government of Tremp (Spain) gave permission to execute our study without any restrictions.

We included apparently healthy adults. Exclusion criteria were cardiovascular diseases, psychiatric diseases, and chronic use of medication for serious illnesses.

### 2.2. Study Design

Groups of 11 subjects, at most, participated in this 10-day trip through the Spanish Pyrenees during the summers of 2011 (*n* = 10), 2012 (*n* = 32), and 2013 (*n* = 11). The participants lived outdoors and walked from one water-source to another. Food was provided by the organization and with help of forest-guards from official institutes of the Catalan county. Food intake was planned before the trip, based on the average daily food intake by the traditionally living Hadzabe people in Tanzania (Supplementary Tables 1 and 2 in Supplementary Material available online at http://dx.doi.org/10.1155/2016/6935123). The use of mobile phones or other electronic devices was not allowed.

The detailed activities and condition during the 10-day study were as follows:

(i) The first day was the day of arrival at the hospital of Tremp (Catalonia, Spain) with an air-conditioned bus from Barcelona (2.5 h drive). Participants were once again informed about the trip. Anthropometric data and blood samples were collected in the fasting state.
(ii) There were daily walking trips from waterhole to waterhole, with an average walking distance of about 14 km/day, including altitude differences up to approximately 1,000 m. The participants carried their own backpacks with an average weight of 8 kg. The trip took place in the part of the Pre-Pyrenees with a maximum altitude of 1,900 meters above sea level.
(iii) Participants consumed two meals daily. The first meal was provided by the organization halfway and the second meal prepared on arrival at the camping site. Animals, including ducks, chickens, turkeys, rabbits, and fish, were delivered alive and killed by the participants. Fish were caught with nets in the Noguera river. All foods were prepared on the spot by the participants. Nutritional details are in Supplemental Table 2.
(iv) The participants slept outside in sleeping bags on small inflatable mattresses. Outside temperatures varied from 22 to 42°C during daylight, whereas night temperatures varied from 12 to 21°C. One group experienced a day of snow in the middle of July, which prompted the organization to provide hotel accommodations for a single night.
(v) Bulk (intermittent) drinking behavior was recommended by drinking as much as possible (up to satiety) after reaching a waterhole. The waterholes contained nonchloritized drinking water.
(vi) Some manual work was done to clean mountain trails as agreed upon with the Catalan Government.

### 2.3. Anthropometric Data, Sample Collection, and Analyses

Sixteen anthropometric and clinical chemical parameters were measured before departure to the Pyrenees and after return. Anthropometric measurements were measured by one of us (LP) at the hospital of Tremp. Fasting heparin-, oxalate-, and EDTA-anticoagulated blood samples were collected by venipuncture in the first morning and the morning of the 10th day at the study end. All samples were transported at 5°C and processed within 1 h. Analyses were done in the clinical laboratory of the hospital of Tremp.

 HbA1c, total cholesterol, HDL-cholesterol, aspartate aminotransferase (ASAT), alanine aminotransferase (ALAT), and glucose were determined with a Cobas c-501 analyzer (Roche, Madrid, Spain). Serum insulin was measured by chemiluminescent assays, using Dxi-600 (Beckman, Barcelona, Spain) and Liaison XL (Diasorin, Madrid, Spain), respectively. High sensitivity C-reactive protein (CRP) was measured with a Behring Nephelometer II Analyzer System. HOMA-IR (mmol*∗*mU/L^2^) was calculated by glucose (in mmol/L)*∗*insulin/22.5 (in mU/L).

### 2.4. Statistics

Statistical analyses were performed with IBM SPSS Statistics version 23.0 (IBM Corp.). Changes during the intervention were analyzed with the Wilcoxon signed rank test at *p* < 0.05. Interrelations between variables were analyzed by Spearman's correlation coefficient at *p* < 0.05.

## 3. Results

### 3.1. Study Group

There were 55 participants. Their median age was 38 years (range 22–67). The number of women was 28 (50.9%). Baseline anthropometrics are in Table 1.

### 3.2. Course of the Trip

The 10-day trip went through the low and middle-high part of the Pyrenees. The majority of participants did very well. If one of them got too tired, LP or JA carried his/her backpack as long as needed. Trips were made from one waterhole to another, with a consistent midday pause of 1–1.5 h. The mountainside of the Pyrenees is composed of hard soil and much of its vegetation carries thorns. Most participants suffered from small wounds on the arms and legs, caused by the thorns of the aliaga, a plant belonging to the original vegetation of the Pyrenees. None of them suffered from infected wounds and most appeared fully cured at the end of the trip. Participants suffered from hunger feelings during the first three days but gradually got used to eating only twice daily. Only one participant discontinued the trip, because she had different expectations. The local organization arranged transport and she left. The other participants enjoyed the trip for the full 10 days, without exceptions. Interestingly, the majority, including LP, wanted to go home after 7 days (see Section 4).

Feelings of thirst were moderate at the beginning but ameliorated after 3 days. Participants noticed that they could drink increasingly more water upon arrival at a waterhole and gradually experienced less needs for “in-between water stops.” Three participants suffered from what might have been neuroglycopenic periods, feeling weak, hungry, cold, dizzy, and trembling. However, measurement of their blood glucose level by finger prick did not reveal hypoglycemia. One of these participants was grossly overweight and exhibited fasting glucose highly surpassing the normal range. He was not able to carry his backpack during the first three days but managed surprisingly well during the last. At night, some participants were affected by the cold. They needed a thicker sleeping bag, which was supplied. Participants went asleep at sunset and rose at sunrise. Overall, participants felt good, occasionally tired, but not at all overloaded. Mosquitoes were a nuisance at night and therefore a liquid mosquito repellent was provided by the organization. Several participants suffered from diarrhea at the trip's end, probably from drinking water from the nonchloritized waterholes. Taken together, the vast majority of the participants enjoyed the trip and recognized the benefits by feeling healthier and recovered from Western stressful life.

### 3.3. Anthropometrics and Clinical Chemical Indices

Anthropometric and clinical chemical data collected before and after the excursion were available from 23–55 participants (Table 1). The missing values were attributable to the 2012 groups. Probably because of procedural imperfections in the Tremp lab, the outcome of the insulin assay could not be performed in various series.

We found (Table 1) that body weight decreased with a median (range) of −3.8 kg (−12.5 to −0.7), BMI with −1.2 kg/m^2^ (−4.4 to −0.2), hip circumference with −3 cm (−17 to +5), waist circumference with −5 cm (−18 to +9), and waist/hip ratio with −0.02 (−0.14 to +0.10).

We also observed decreases (median; range; Table 1) of glucose (−0.6; −1.7 to +0.5 mmol/L), HbA1c (−0.1; −0.4 to +0.2%), insulin (−4.7; −31.4 to −0.2 pmol/L), HOMA-IR (−1.2; −7.0 to −0.4 mmol*∗*mU/L^2^), triglycerides (−0.14; −6.12 to +2.18 mmol/L), total cholesterol (−0.7; −2.8 to +0.4 mmol/L), LDL-cholesterol (−0.6; −3.1 to +0.6 mmol/L), triglycerides/HDL-cholesterol ratio (−0.55; −8.98 to 1.34 mol/mol), and FT3 (−0.8; −3.4 to +3.1 pmol/L).

**Figure 1:**
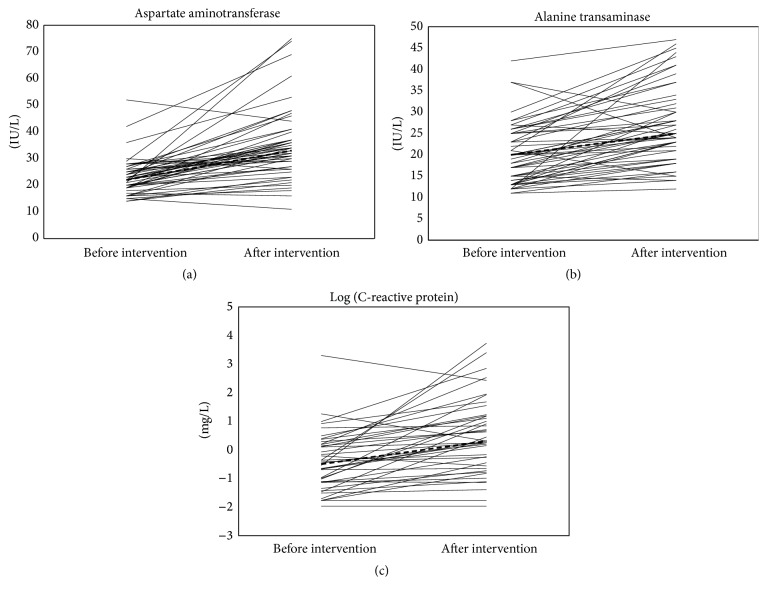
Median and individual changes of ASAT (a), ALAT (b), and CRP (c) during the 10-day trip through the Pyrenees.

On the other hand we found that ASAT and ALAT activities increased with 11 IU/L (−8 to 54) and 6 IU/L (−13 to 52), respectively, while CRP increased with 0.56 mg/L (−15.72 to +41.07).  Figure 1 shows the median and individual changes of ASAT (a), ALAT (b), and CRP (c). The ASAT/ALAT ratio before the intervention was 1.23 (0.68–2.00) and increased with 0.08 to 1.31 (0.48–2.06).

**Figure 2:**
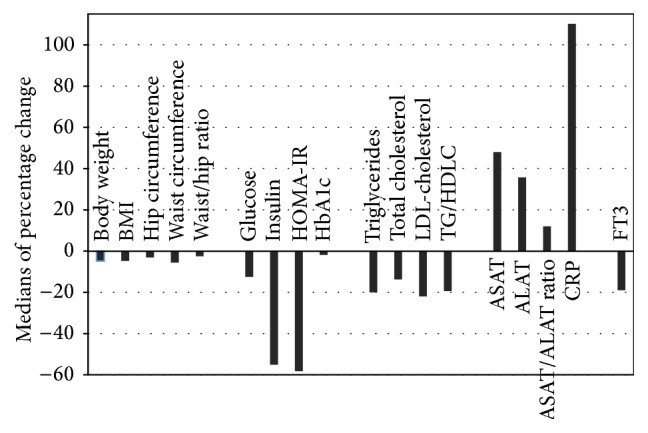
Medians of the percentage changes of anthropometric and clinical chemical parameters during the 10-day trip through the Pyrenees. Only significant changes are shown (see Table 1). For abbreviations see the footnote of Table 1.

Figure 2 shows the medians of the percentage change for the anthropometric and clinical chemical parameters that were found to change significantly during the 10-day trip through the Pyrenees.

### 3.4. One Subject with Arrhythmia

One of the participants suffered from cardiac arrhythmia since 2000, following a sternum fracture in a car accident. He was on medication since then. During the trip he stopped taking treatment and did not suffer from arrhythmic periods during the 36-month period that passed since the study end.

### 3.5. Interrelationships

Relationships between changes of the anthropometric and clinical chemical indices are presented in Supplemental Table 3. Importantly, we found that the changes in weight, BMI, hip circumference, waist circumference, and waist/hip ratio were generally unrelated to the changes of clinical chemical indices. Exceptions were the negative associations (*p* < 0.05) between waist circumference and FT3 (*r* = −0.359) and waist/hip ratio and FT4 (*r* = −0.360). The change in HOMA-IR was negatively related to changes in total cholesterol (*r* = −0.457) and LDL-cholesterol (*r* = −0.436). Finally, we found that the changes in ASAT, ALAT, and CRP were positively interrelated (ASAT versus ALAT *r* = 0.777; ASAT versus CRP *r* = 0.508; and ALAT versus CRP *r* = 0.440). Age proved positively related to the change of ASAT (*r* = 0.371) but was unrelated to the changes of ALAT and CRP.

## 4. Discussion

The aim of this study was to investigate whether a 10-day trip through the Pyrenees favorably affects anthropometric-, metabolic-, and inflammatory-parameters in apparently healthy subjects. The trip mimicked to some extent the “conditions of existence” of ancient and contemporary hunting-gathering populations. We found that the intervention was well tolerated and that all participants, except for one dropout, experienced a better subjective feeling of health following its completion. The trip caused decreases in body weight and BMI (median change 4.8%), hip circumference (3%), waist circumference (5.6%), and waist/hip ratio (2.5%) (Table 1; Figure 2). Among the clinical chemical indices we found decreases of glucose (12.5%), insulin (55%), HOMA-IR (58.1%), HbA1c (1.8%), triglycerides (20%), total cholesterol (13.7%), LDL-cholesterol (21.9%), and triglycerides/HDL-cholesterol ratio (19.3%). On the other hand, the medians of ASAT and ALAT increased with 48.0% and 35.7%, respectively, while CRP increased with 110.1%.

### 4.1. Favorable Effects

Altogether we found that 3 features of the metabolic syndrome improved, that is, body mass, glucose homeostasis, and circulating lipids. The fourth, that is, blood pressure, was not recorded. The metabolic syndrome, also named the insulin resistance syndrome, is a well-established risk factor for various diseases of affluence, including type 2 diabetes, cardiovascular disease, essential hypertension, polycystic ovary syndrome, nonalcoholic fatty liver disease, certain types of cancer (colon, breast, and pancreas), sleep apnea, and pregnancy complications, such as preeclampsia and gestational diabetes [32]. Although we did not aim at hard endpoints, our results suggest that the 10-day intervention could be of value to both primary and secondary prevention of the “typically Western diseases of affluence.”

### 4.2. Potential Mechanisms

Our intervention is based on causing “mild acute stress” in humans who in their usual daily lives are exposed to the chronic stress commensurate with our modern lifestyle. Acute stress promotes release of stress hormones, including adrenaline, noradrenaline, and cortisol, that each cause profound metabolic and immunologic adaptations [33]. For instance, a recent study by the group of Pickkers [34] showed that extreme cold exposure, combined with breathing exercise (producing intermittent hypoxia), profoundly increases adrenaline secretion. This study and others [33, 35] show that acute stress factors increase autonomic activity, accelerate immune cell proliferation and differentiation, and also stimulate the anti-inflammatory component of the immune system (i.e., production of IL10, lactoferrin, and lysozymes) [26]. Nevertheless, mild stress initially produces a proinflammatory response, which may subsequently give rise to recovery from the reigning state of chronic low-grade inflammation and the return to homeostasis [36, 37].

In line with the above, we found that the observed changes in HOMA-IR and lipids were independent of weight loss, suggesting that a combination of lifestyle factors might be at stake. Major, highly interacting, lifestyle factors contributing to typically Western diseases are poor diet, insufficient physical activity, chronic stress, insufficient sleep, abnormal microbial flora, and environmental pollution (smoking included) [11, 38]. However, other mismatches with our ancient lifestyle are less appreciated. For instance, the participants suffered from thirst. Thirst relates to oxytocin production and the inhibition of stress axis activity [39, 40]. The participants were also disconnected from daily trouble and “self-made” stress, which reduced the number of anthropogenic stress factors and possibly other inflammatory “danger signals” [16]. Another factor might be the prohibited use of mobile telephones and other electronic devices. Although controversial, chronic mobile telephone usage may activate stress systems as evidenced by a recent study of Hamzany et al. [41]. Chronic use of mobile telephones also negatively affects the production of anti-inflammatory substances in saliva [42]. Absence of artificial light might be another factor. The trip forced the participants to adopt a “natural day-night rhythm” in which the sleep-wake cycle was not dominated by social life, but rather by sunlight [43–45].

Spontaneous physical activity prior to food and water intake might be another beneficial factor. The postprandial inflammatory response has been identified as an independent risk factor for cardiovascular, metabolic, and other noncommunicable disorders [26, 46–48]. Preprandial exercise not only blunted the proinflammatory response after food intake, but also produced a shift towards the production of anti-inflammatory mediators by the immune system and adipose tissue, conferring protection against possible pathogens present in food [49, 50]. Another important difference between Western life and the 10-day trip in the Pyrenees might be the presence of “cutaneous- and other body surface-directed danger signals.” All participants suffered from little wounds on the hands, arms, and legs because of small injuries inflicted by sharp thorns and other natural obstacles. In addition, several participants suffered from mild gastrointestinal disorders and diarrhea. Fiuza-Luces et al. [51] attributed positive health effects to the so-called hormetic triggers, including small external wounds and light muscle damage. Although speculative, the immune system might have migrated to sites that have been most susceptible to the damaging effects of the environment during evolution, including those affecting the skin, the gastrointestinal tract, and the lungs, jointly being the sites with the highest need of immune surveillance [33].

In summary, we propose that we are dealing with complex interacting lifestyle factors [5, 11] and that the current tendency to perform interventions aiming at single components, whether or not designed as RCTs, might suffer from a reductionist approach.

### 4.3. Possible Adverse Effects

We found increases of ASAT, ALAT, and CRP, which at first glance might be regarded as genuine adverse effects. Increases of ASAT and the ASAT/ALAT ratio are related to cardiac muscle damage [52], while those of ALAT [53] and CRP [54] are intimately related to liver damage and infection, respectively.

Extreme sports, such as marathons and triathlons, are well known to elicit increases of lactate dehydrogenase, creatine kinase, ALAT, ASAT, ASAT/ALAT ratio, CRP, and cardiac troponins [55–58]. The responses may easily exceed the upper limit of the reference range into the myocardial infarction area. The increases of cardiac troponins were similar when exercise was performed under controlled normoxic or hypoxic conditions but proved dependent on exercise duration and intensity, possibly aggravated by hypoxic conditions [56]. Noakes and Carter [59] also observed that novice runners had much higher responses than experienced runners and these results were confirmed in a later study [60]. We found that the augmentation of ASAT increased with age, as previously reported by Jastrzębski et al. [57]. Increases of cardiac markers under these conditions have not been firmly associated with irreversible cardiac muscle damage and are at present considered benign, at least in well-trained subjects. On the other hand, there are currently no data from long-term follow-ups [61].

The increases of both ALAT and CRP might also point at moderate liver damage and inflammation due to environmental exposure, the latter causing mild gastrointestinal infections from drinking water from waterholes, and small injuries on the legs and arms inflicted by thorns and falls. Although the aforementioned plausible adverse effects will definitely require more attention in future interventions, they are not unexpected in the light of hunter-gatherer populations. For instance, the Pygmies exhibit huge gamma-globulin bands in the classical electrophoretic profiling of serum proteins, pointing at exposure to a host of different microorganisms and parasites [62]. Therefore, apart from the effects of intensive physical activity, the current findings remind us of the super-hygienic conditions of our current lifestyle. Hygiene is a major factor in longevity but may, as a trade-off, also be at the basis of, for example, autoimmune disease, the so-called hygiene hypothesis [63–65]. For instance, a recent study in pregnant and newborn mice revealed that helminth colonization exerts beneficial effects on the infectious response of the offspring brain and also on microglial sensitization and cognitive dysfunction in adult life [66].

### 4.4. Comparison with Previous Studies

Our study is not the first to suggest that mimicking the lifestyles of traditional hunter-gatherer populations may be beneficial to our health. Already in 1984, O'Dea et al. showed that overweight Australian Aborigines with type 2 diabetes who reverted to their original lifestyle for 7 weeks were able to improve, or even normalize, the characteristic abnormalities of diabetes, including improvements of body weight and fasting glucose, insulin, and triglycerides. The favorable changes were attributed to weight loss, low-fat diet, and increased physical activity [67]. Other examples of the protective effects of our “ancient conditions of existence” against modern lifestyle and “Western diseases of affluence” may be compiled from the studies of the Kitava people in Papua New Guinea [68, 69]. Lindeberg et al. showed that these people, living a traditional lifestyle (e.g., consuming wild foods with profound physical activity), showed low rates of cardiovascular disease, obesity, and other modern diseases, probably because of higher insulin sensitivity and lower levels of insulin, uric acid, and leptin [70].

## 5. Limitations

Our study has many limitations. There was no control group and we also did not employ a crossover or RCT-like design. The investigated group was relatively small and we only measured a small number of “soft” parameters. Hard endpoints can obviously not be expected in short-term interventions in small groups with good general health. However, whether a placebo effect or not, the subjective feeling of better health is certainly important and so are the observed changes in anthropometric and clinical chemical parameters. Our primary objective was to provide a proof of principle. More studies with more participants are certainly needed, including the recording of more parameters to objectivize, for example, the feeling of improved health. Also the minimum duration and intensity needed to demonstrate favorable effects are uncertain as yet.

## 6. Conclusions

The outcome of this 10-day “study of origin” suggests that a short period of return to the “conditions of existence” similar to those on which our genome is based may improve anthropometrics and metabolism by favorably challenging the immune system in apparently healthy subjects. The “return” may come with some costs in more active infection, as a trade-off for the “chronic systemic low-grade inflammation” typical of our current lifestyle of affluence. We may increasingly appreciate that we cannot have it all, while the evolutionary lessons of Darwin and intervention studies [71] teach us that prevention might be more rewarding and affordable than the current culture of medical treatment.

## Supplementary Material

Food consumed during the 10 day trip (table S2) and based on the food intake of current hunter-gatherer populations (table S1). Correlations between all measured parameters (table S3).

## Figures and Tables

**Table 1:** Anthropometrics and clinical chemical indices at baseline and at the study end.

	Unit	*N*	Baseline		Study end		Change				*p* value
			Median	Range	Median	Range	Median	Range	SD	95% CI of the mean	
Body weight	kg	55	68.0	48.4–116.3	65.00	46.9–111.8	−3.8	−12.5 to −0.7	2.0	−4.4 to −3.3	<0.001^*∗*^
Age	Years	50	38	22–67	NM	NM	NM	NM	NM	NM	NM
Height	cm	55	175	154–203	NM	NM	NM	NM	NM	NM	NM
BMI	kg/m^2^	55	22.40	17.4–31.9	21.3	16.8–30.4	−1.2	−4.4 to −0.2	0.6	−1.4 to −1.1	<0.001^*∗*^
Hip circumference	cm	44	100	85–120	96	86–115	−3	−17 to 5	3.3	−4.2 to −2.2	<0.001^*∗*^
Waist circumference	cm	44	81	66–110	76	63–101	−5	−18 to 9	5.5	−7 to −4	<0.001^*∗*^
Waist/hip ratio	cm/cm	44	0.84	0.72–1.00	0.80	0.66–0.94	−0.02	−0.14 to 0.10	0.06	−0.04 to −0.02	0.002^*∗*^

Glucose	mmol/L	53	4.9	4.2–5.8	4.3	3.3–6.1	−0.6	−1.7 to 0.5	0.6	−0.8 to −0.5	<0.001^*∗*^
HbA1c	%	53	5.3	4.8–6.1	5.3	4.7–6.1	−0.1	−0.4 to 0.2	0.2	−0.1 to −0.05	<0.001^*∗*^
Insulin	mU/L	23	14.0	3.7–36.8	6.7	1.1–12.9	−4.7	−31.4 to −0.2	8.1	−12.2 to −5.2	<0.001^*∗*^
HOMA-IR	mmol**∗**mU/L^2^	22	3.0	0.8–7.9	1.4	0.2–2.6	−1.2	−7.0 to −0.4	1.8	−2.8 to −1.3	<0.001^*∗*^

Triglycerides	mmol/L	53	0.69	0.34–6.68	0.52	0.37–2.77	−0.14	−6.12 to 2.18	0.92	−0.52 to −0.01	<0.001^*∗*^
Total cholesterol	mmol/L	53	5.2	3.2–8.2	4.5	2.6–8.1	−0.7	−2.8 to 0.4	0.7	−1.0 to −0.6	<0.001^*∗*^
HDL-cholesterol	mmol/L	53	2.0	0.7–3.1	1.9	1.0–3.5	0.0	−0.8 to 0 .6	0.3	−0.1 to 0.1	0.464
LDL-cholesterol	mmol/L	52	3.0	1.3–5.8	2.5	0.0–5.4	−0.6	−3.1 to 0.6	0.7	−0.8 to −0.5	<0.001^*∗*^
TG/HDL-cholesterol ratio	mol/mol	53	0.3	0.16–9.54	0.26	0.11–1.73	−0.55	−8.98 to 1.34	1.3	−0.59 to 0.98	<0.001^*∗*^

ASAT	IU/L	53	22	14–52	33	11–75	11	−8 to 54	11.4	9 to 15	<0.001^*∗*^
ALAT	IU/L	53	20	11–42	25	12–47	6.0	−13 to 52	7.3	5 to 9	<0.001^*∗*^
CRP	mg/L	42	0.61	0.14–27.04	1.36	0.14–41.65	0.56	−15.72 to 41.07	8.45	0.20 to 5.46	<0.001^*∗*^

TSH	mU/L	42	1.25	0.02–3.12	1.11	0.02–4.40	−0.08	−0.93 to 1.28	0.47	−0.19 to −0.10	0.326
FT4	pmol/L	42	10.8	7.9–19.4	11.3	7.8–20.6	0.1	−5.6 to 8.4	2.3	−0.4 to 1.1	0.378
FT3	pmol/L	42	4.4	2.3–6.5	3.5	1.7–8.7	−0.8	−3.4 to 3.1	1.0	−1.0 to −0.5	<0.001^*∗*^

Data are medians (range). ALAT, alanine aminotransferase; ASAT, aspartate aminotransferase; BMI, body mass index; CRP, C-reactive protein; FT3, free triiodothyronine; FT4, free thyroxine; HbA1c, hemoglobin A1c; HDL, high-density lipoprotein; HOMA-IR, homeostasis model assessment-estimated insulin resistance; LDL, low-density lipoprotein; NM, not measured; TG, triglycerides; TSH, thyroid-stimulating hormone. ^*∗*^Significant difference between the values before and after the intervention by Wilcoxon signed rank test at *p* < 0.05.
