# Corrigendum to “Influence of a 10-Day Mimic of Our Ancient Lifestyle on Anthropometrics and Parameters of Metabolism and Inflammation: The “Study of Origin””

**DOI:** 10.1155/2017/1641589

**Published:** 2017-03-13

**Authors:** Leo Pruimboom, Begoña Ruiz-Núñez, Charles L. Raison, Frits A. J. Muskiet, Jens Freese

**Affiliations:** ^1^Natura Foundation, 3281 NC Numansdorp, Netherlands; ^2^Laboratory Medicine, University Medical Center Groningen (UMCG) and University of Groningen, 9713 GZ Groningen, Netherlands; ^3^Department of Psychiatry, College of Medicine, John and Doris Norton School of Family and Consumer Sciences, Tucson, AZ 85719, USA; ^4^German Sports University Cologne, Cologne, Germany

In the article titled “Influence of a 10-Day Mimic of Our Ancient Lifestyle on Anthropometrics and Parameters of Metabolism and Inflammation: The “Study of Origin”” [1], Jens Freese was missing from the authors' list. The corrected authors' list is shown above.

Also, there was a typographical error in the value of body weight loss. As a result, in the second paragraph of “Anthropometrics and Clinical Chemical Indices” section, the sentence “We found (Table 1) that body weight decreased with a median (range) of −3.8 kg (−12.5 to −0.7)” should be changed to “We found (Table 1) that body weight decreased with a median (range) of −3.4 kg (−7.5 to −0.7),” and Table 1 should be corrected as follows:

## Figures and Tables

**Table 1:** Anthropometrics and clinical chemical indices at baseline and at the study end.

	Unit	*N*	Baseline		Study end		Change				*p* value
			Median	Range	Median	Range	Median	Range	SD	95% CI of the mean	
Body weight	kg	55	68.0	48.4–116.3	65.00	46.9–111.8	−3.4	−7.5 to −0.7	2.0	−4.4 to −3.3	<0.001^*∗*^
Age	Years	50	38	22–67	NM	NM	NM	NM	NM	NM	NM
Height	cm	55	175	154–203	NM	NM	NM	NM	NM	NM	NM
BMI	kg/m^2^	55	22.40	17.4–31.9	21.3	16.8–30.4	−1.2	−4.4 to −0.2	0.6	−1.4 to −1.1	<0.001^*∗*^
Hip circumference	cm	44	100	85–120	96	86–115	−3	−17 to 5	3.3	−4.2 to −2.2	<0.001^*∗*^
Waist circumference	cm	44	81	66–110	76	63–101	−5	−18 to 9	5.5	−7 to −4	<0.001^*∗*^
Waist/hip ratio	cm/cm	44	0.84	0.72–1.00	0.80	0.66–0.94	−0.02	−0.14 to 0.10	0.06	−0.04 to −0.02	<0.002^*∗*^

Glucose	mmol/L	53	4.9	4.2–5.8	4.3	3.3–6.1	−0.6	−1.7 to 0.5	0.6	−0.8 to −0.5	<0.001^*∗*^
HbA1c	%	53	5.3	4.8–6.1	5.3	4.7–6.1	−0.1	−0.4 to 0.2	0.2	−0.1 to −0.05	<0.001^*∗*^
Insulin	mU/L	23	14.0	3.7–36.8	6.7	1.1–12.9	−4.7	−31.4 to −0.2	8.1	−12.2 to −5.2	<0.001^*∗*^
HOMA-IR	mmol*∗*mU/L^2^	22	3.0	0.8–7.9	1.4	0.2–2.6	−1.2	−7.0 to −0.4	1.8	−2.8 to −1.3	<0.001^*∗*^

Triglycerides	mmol/L	53	0.69	0.34–6.68	0.52	0.37–2.77	−0.14	−6.12 to 2.18	0.92	−0.52 to −0.01	<0.001^*∗*^
Total cholesterol	mmol/L	53	5.2	3.2–8.2	4.5	2.6–8.1	−0.7	−2.8 to 0.4	0.7	−1.0 to −0.6	<0.001^*∗*^
HDL-cholesterol	mmol/L	53	2.0	0.7–3.1	1.9	1.0–3.5	0.0	−0.8 to 0.6	0.3	−0.1 to 0.1	0.464
LDL-cholesterol	mmol/L	52	3.0	1.3–5.8	2.5	0.0–5.4	−0.6	−3.1 to 0.6	0.7	−0.8 to −0.5	<0.001^*∗*^
TG/HDL-cholesterol ratio	mol/mol	53	0.3	0.16–9.54	0.26	0.11–1.73	−0.55	−8.98 to 1.34	1.3	−0.59 to 0.98	<0.001^*∗*^

ASAT	IU/L	53	22	14–52	33	11–75	11	−8 to 54	11.4	9 to 15	<0.001^*∗*^
ALAT	IU/L	53	20	11–42	25	12–47	6.0	−13 to 52	7.3	5 to 9	<0.001^*∗*^
CRP	mg/L	42	0.61	0.14–27.04	1.36	0.14–41.65	0.56	−15.72 to 41.07	8.45	0.20 to 5.46	<0.001^*∗*^

TSH	mU/L	42	1.25	0.02–3.12	1.11	0.02–4.40	−0.08	−0.93 to 1.28	0.47	−0.19 to −0.10	0.326
FT4	pmol/L	42	10.8	7.9–19.4	11.3	7.8–20.6	0.1	−5.6 to 8.4	2.3	−0.4 to 1.1	0.378
FT3	pmol/L	42	4.4	2.3–6.5	3.5	1.7–8.7	−0.8	−3.4 to 3.1	1.0	−1.0 to −0.5	<0.001^*∗*^

Data are medians (range). ALAT, alanine aminotransferase; ASAT, aspartate aminotransferase; BMI, body mass index; CRP, C-reactive protein; FT3, free triiodothyronine; FT4, free thyroxine; HbA1c, hemoglobin A1c; HDL, high-density lipoprotein; HOMA-IR, homeostasis model assessment-estimated insulin resistance; LDL, low-density lipoprotein; NM, not measured; TG, triglycerides; TSH, thyroid-stimulating hormone. ^*∗*^Significant difference between the values before and after the intervention by Wilcoxon signed rank test at *p* < 0.05.
