# Supplementary material for: Influence of a 10-Day Mimic of Our Ancient Lifestyle on Anthropometrics and Parameters of Metabolism and Inflammation: The “Study of Origin”
Source: Biomed Res Int. 2016 Jun 6;2016:6935123. doi: 10.1155/2016/6935123 (PMC4913061; doi:10.1155/2016/6935123)
Supplement: Supplementary file 1 — Food consumed during the 10 day trip (table S2) and based on the food intake of current hunter-gatherer populations (table S1). Correlations between all measured parameters (table S3). [file 6935123.f1.docx]

**Supplemental Table 1.** Average food intake of Hadzabe hunter-gatherers

| Food consumed | Hadza en% | kcal |
| --- | --- | --- |
| Meat | 25 | 625 |
| Tubers | 17 | 425 |
| Wild Berries | 25 | 625 |
| Fruits |  |  |
| Eggs |  |  |
| Fish |  |  |
| Nuts |  |  |
| Green  Vegetables |  |  |
| Baobab | 11 | 275 |
|  |  |  |
| Honey | 22 | 550 |
|  |  |  |
|  |  |  |
|  |  |  |
| Total | 100 | 2500 |
|  |  |  |

Data derive from Pontzer 2012, Murray 2001, IDF 2012, [www.philosophy.dept.shef.ac.uk/culture&mind/people/crittendena/](http://www.philosophy.dept.shef.ac.uk/culture&mind/people/crittendena/)

1. Murray, S. S., Schoeninger, M. J., Bunn, H. T., Pickering, T. R. & Marlett, J. A. Nutritional Composition of Some Wild Plant Foods and Honey Used by Hadza Foragers of Tanzania. *Journal of Food Composition and Analysis* **14,** 3-13 (2001).

2. Pontzer, H., Raichlen, D. A., Wood, B. M., Mabulla, A. Z., *et al.* Hunter-gatherer energetics and human obesity. *PLoS One* **7,** e40503 (2012).

**Supplemental Table 2.** Average food intake of participants during the ten days trip through the Pyrenees (amount/10 persons)

| **Nutrient** | **Average amount** | **kcal /unit** | **total kcal** | **kcal/part**  **/10 days** |
| --- | --- | --- | --- | --- |
|  |  |  |  |  |
| Banana | 80 | 105 | 8400 | 840 |
| Apple | 70 | 20 | 1400 | 140 |
| Melon | 10 kilo | 300 | 3000 | 300 |
| Mangos | 4,5 kilo | 645 | 2900 | 290 |
| Pineapple | 3 kilo | 460 | 1380 | 138 |
| Watermelon | 8 kilo | 280 | 2260 | 226 |
| Dates | 1,1 kilo | 2760 | 3036 | 304 |
| Onions | 12 kilo | 400 | 4800 | 480 |
| Lettuce (6 types) | 3 kilo | 160 | 480 | 48 |
| Cucumber | 3 kilo | 112 | 336 | 33 |
| Garlic | 2 kilo | 132 | 264 | 26 |
| Olives | 1,5 kilo | 1660 | 2500 | 250 |
| Carrots | 2,4 kilo | 410 | 985 | 99 |
| Mushrooms | 1,1 kilo | 225 | 250 | 25 |
| Zucchini | 1,5 kilo | 160 | 240 | 24 |
| Asparagus | 1 kilo | 220 | 220 | 22 |
| Avocado | 6 kilo | 1650 | 10100 | 1010 |
| Pumpkin | 2 kilo | 200 | 400 | 40 |
| Leek | 2 kilo | 550 | 1100 | 110 |
| Sweet potato | 1,5 kilo | 720 | 1080 | 108 |
| Coconut | 5 kilo | 3180 | 15900 | 159 |
| Raisins | 1 kilo | 2860 | 2860 | 286 |
| Berries | 3 kilo | 480 | 1440 | 144 |
|  |  |  | Subtotal | 6532 |
|  |  |  |  |  |
| Mayonaise | 1 kilo | 6300 | 6300 | 630 |
| Olive oil | 3 liters | 8400 | 25200 | 2520 |
| Honey | 2 kilo | 7000 | 14000 | 140 |
|  |  |  | Subtotal | 3290 |
|  |  |  |  |  |
| Deer | 11 kilo | 1480 | 26280 | 2628 |
| Chicken | 9 kilo | 2140 | 19260 | 1920 |
| Rabbit | 2 kilo | 2764 | 5528 | 553 |
| Sweat water fish | 8 kilo | 1590 | 12720 | 1272 |
| Tuna in olive oil | 5 kilo | 4030 | 20150 | 2015 |
| Duck | 6 kilo | 2016 | 12096 | 1209 |
| Eggs | 5,5 kilo | 1400 | 7700 | 770 |
| Nuts | 3,7 kilo | 575 | 20275 | 2027 |
|  |  |  |  |  |
|  |  |  |  |  |
|  |  |  | Subtotal | 12394 |
|  |  |  |  |  |
|  |  |  | Total/pp/trip | 22216 |
|  |  |  | Total/pp/day | 2222 |

**Supplemental Table 3.** Spearman's correlation coefficients for the relations between the changes in anthropometric and clinical chemical indices during the study

Changes refer to the difference between pre- and post-intervention data. Abbreviations: ALAT, alanine aminotransferase; ASAT, aspartate aminotransferase; CRP, C-reactive protein; FT3, free triiodothyronine; FT4, free thyroxine; HBA1c, hemoglobin A1c; HDL, high-density-lipoprotein; HDL-C, high-density-lipoprotein-cholesterol; LDL, low-density lipoprotein; ; N.M. Not measured; TG, triglycerides; TSH, thyroid-stimulating hormone. *, Significant at p<0.05; ** , significant at p<0.01.
